# Transforming RNA-Seq Data to Improve the Performance of Prognostic Gene Signatures

**DOI:** 10.1371/journal.pone.0085150

**Published:** 2014-01-08

**Authors:** Isabella Zwiener, Barbara Frisch, Harald Binder

**Affiliations:** 1 Center for Thrombosis and Hemostasis (CTH), University Medical Center Mainz, Mainz, Germany; 2 Institute of Medical Biostatistics, Epidemiology and Informatics (IMBEI), University Medical Center Mainz, Mainz, Germany; Queen's University Belfast, United Kingdom

## Abstract

Gene expression measurements have successfully been used for building prognostic signatures, i.e for identifying a short list of important genes that can predict patient outcome. Mostly microarray measurements have been considered, and there is little advice available for building multivariable risk prediction models from RNA-Seq data. We specifically consider penalized regression techniques, such as the lasso and componentwise boosting, which can simultaneously consider all measurements and provide both, multivariable regression models for prediction and automated variable selection. However, they might be affected by the typical skewness, mean-variance-dependency or extreme values of RNA-Seq covariates and therefore could benefit from transformations of the latter. In an analytical part, we highlight preferential selection of covariates with large variances, which is problematic due to the mean-variance dependency of RNA-Seq data. In a simulation study, we compare different transformations of RNA-Seq data for potentially improving detection of important genes. Specifically, we consider standardization, the log transformation, a variance-stabilizing transformation, the Box-Cox transformation, and rank-based transformations. In addition, the prediction performance for real data from patients with kidney cancer and acute myeloid leukemia is considered. We show that signature size, identification performance, and prediction performance critically depend on the choice of a suitable transformation. Rank-based transformations perform well in all scenarios and can even outperform complex variance-stabilizing approaches. Generally, the results illustrate that the distribution and potential transformations of RNA-Seq data need to be considered as a critical step when building risk prediction models by penalized regression techniques.

## Introduction

RNA-Seq is a relatively new approach for measuring gene expression by making use of next generation sequencing technology. It produces count data having low background noise and hence allows to detect transcripts even at low expression levels and provides a large dynamic range in terms of fold-changes [1], [2]. Furthermore, RNA-Seq can detect and quantify alternative splicing and previously unknown transcripts [3]–[6]. Therefore, RNA-Seq is on its way to replace the microarray technology, which has been widely used in the last decades.

Gene expression measurements from microarrays have often been used for building prognostic gene signatures, i.e. a small set of genes that can predict the clinical outcome of patients. Correspondingly, it is attractive to also use RNA-Seq data for such a task, but the highly skewed nature of the latter might pose difficulties. In the following, we focus on regularized regression techniques for building signatures from RNA-Seq data, as these simultaneously consider all RNA-Seq measurements, can provide automated selection of important genes, and have generally been a popular class of multivariable approaches for microarray gene expression data. For a more general overview of such approaches, see e.g. Binder et al. [7] and for a comparison of the most common methods see Bøvelstad et al. [8] or van Wieringen et al. [9]. We will specifically consider the lasso [10] and componentwise likelihood-based boosting [11], [12] as representative approaches for regularized regression with variable selection.

The aim of this work is to investigate which specific properties of RNA-Seq data, such as skewness, mean-variance dependency and extreme values, influence model building with these approaches. In particular, we systematically investigate transformations of the RNA-Seq measurements to increase the performance of the models, with respect to identification of important genes and prediction performance.

While there is hardly any advice for multivariable regression modeling with RNA-Seq data, a multitude of univariate testing techniques have been developed [13]–[20] and software tools offering in addition graphical evaluations have become available, see e.g. [21]. Most of the methods model the count data using a Poisson or negative-binomial distribution. A main difference between the methods is how they estimate the variances and dispersion parameters, specifically in the context of small sample sizes. Most frequently used methods include edgeR [13] and DESeq [14], both assuming a negative binomial distribution. DESeq includes a variance-stabilizing transformation (VST), to account for the different variances for the individual genes before applying a test for differential expression (DE), that might also be useful as a first step before multivariable modeling. The SAMSeq method introduced by Li and Tibshirani [16] deals with the extreme values using a nonparametric rank test. NOISeq [17] is another approach which uses nonparametric tests on log2-fold changes. Correspondingly, we will also consider rank-based approaches for regularized regression. While we consider ideas from univariate approaches for improving multivariable modeling, we will not consider a comparison of univariate and multivariable approaches, as these two classes of techniques have different aims. For a recent comparison of the most frequently used univariate methods see, e.g., Soneson et al. [22]. Naturally, we cannot exhaustively investigate potential transformations, which even might have been suggested outside RNA-Seq applications. For example, a pre-transformation was proposed by Boulesteix et al. in the context of microarray data [23], and might potentially also be adapted for RNA-Seq data.

Besides transformations, such as using the VST or ranks, the variances of covariates are a critical issue for regularized regression techniques. Often, standardizing gene expressions is implemented as a default in software packages. For other kinds of molecular measurements, such as single nucleotide polymorphism data, standardization has not always be found to be advantageous [24]. Therefore, we also consider the performance implications of standardization for RNA-Seq data. Naturally, standardizing of covariates depends on estimation of variances, which might again be problematic for RNA-Seq data due to the skewness, the mean-variance-dependency of count data, and the presence of extreme values. As the latter issues already might be addressed by the transformations indicated above, we will consider standardization jointly with different kinds of transformation for judging the resulting performance.

In this work we propose a set of different data transformations of RNA-Seq data that can be applied before building a prognostic gene signature for binary endpoints and time-to-event endpoints. Transforming the data can be used to account for mean-variance dependencies and extreme values, both typical for RNA-Seq data. We compare the resulting gene signatures in terms of sensitivity, specificity and prediction performance using a simulation study in which we focus on a binary endpoint and componentwise likelihood-based boosting. As we are not only interested in binary endpoints and boosting, we will apply all transformations on two different real RNA-Seq data sets of patients in which we focus on time-to-event endpoints and boosting as well as the lasso. One data set is from patients with kidney renal clear cell carcinoma and the other from patients with acute myeloid leukemia. In the latter settings, we will use the gene signature to predict the survival times of the patients adjusting for known clinical covariates. Within these application examples we will have a close look on the individual differences of the gene signatures emerging from the different transformations including signature size, variance of selected genes and prediction performance given by the added value compared to a prognostic model only including the clinical covariates.

The rest of this work is organized as follows. We will first introduce the application examples with kidney renal clear cell carcinoma (KIRC) data and acute myeloid leukemia (AML) data to further motivate this work and highlight some important properties of real RNA-Seq data. In the subsequent section we will show analytically how the variance of covariates affects the gene selection process with regularized regression techniques. We then provide a simulation study to investigate the effect of transformations and standardization on identification of genes. The simulation study considers a binary response, e.g. reflecting a two-group setting with a logistic regression model, and is based on the covariate structure of the KIRC application to simulate realistic conditions, including different expression strengths with different variances, skewed data, extreme values and correlations. Following the results of this simulation study we will give detailed results on both real data application to the KIRC and AML data, where time-to-event endpoints with a Cox regression model are considered.

## Materials

### 2.1 Kidney renal clear cell carcinoma (KIRC) data

As a first application example, we consider RNA-Seq data from patients with kidney renal clear cell carcinoma (KIRC), available from The Cancer Genome Atlas (TCGA) project (website: https://tcga-data.nci.nih.gov/tcga/). RNA-Seq data is available for 470 patients. We excluded five patients because their RNA-Seq data were available twice with different expression values, and one further patient due to an unknown survival time. Genes having a maximum number of 10 counts were excluded as they showed almost no expression (625 genes). After this preprocessing, we normalized the raw counts between the patients using the DESeq normalization proposed by Anders and Huber [14]. Genes with unknown gene length were excluded (n = 680), because there might be gene length effects [25] and we need them later in the simulation study. Extreme values were truncated at the median gene expression plus three times the interquartile range per gene, which is similar to the pre-transformation suggested by Boulesteix et al. for microarray data [23]. After these preprocessing and normalization steps we end up with 464 patients with known survival times and RNA-Seq data for 19,227 genes. The median overall survival time for this patient cohort is 6.3 years, 3-year and 5-year overall survival rates are estimated to be 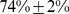 and 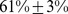. In addition to the survival times there is some clinical information available, e.g. age at diagnosis, sex or tumor stage. This calls for an analysis that can quantify the added value of using RNA-Seq in addition to the clinical characteristics for prognosis, i.e. for a multivariable risk prediction approach, as provided by regularized regression.

**Figure 1:**
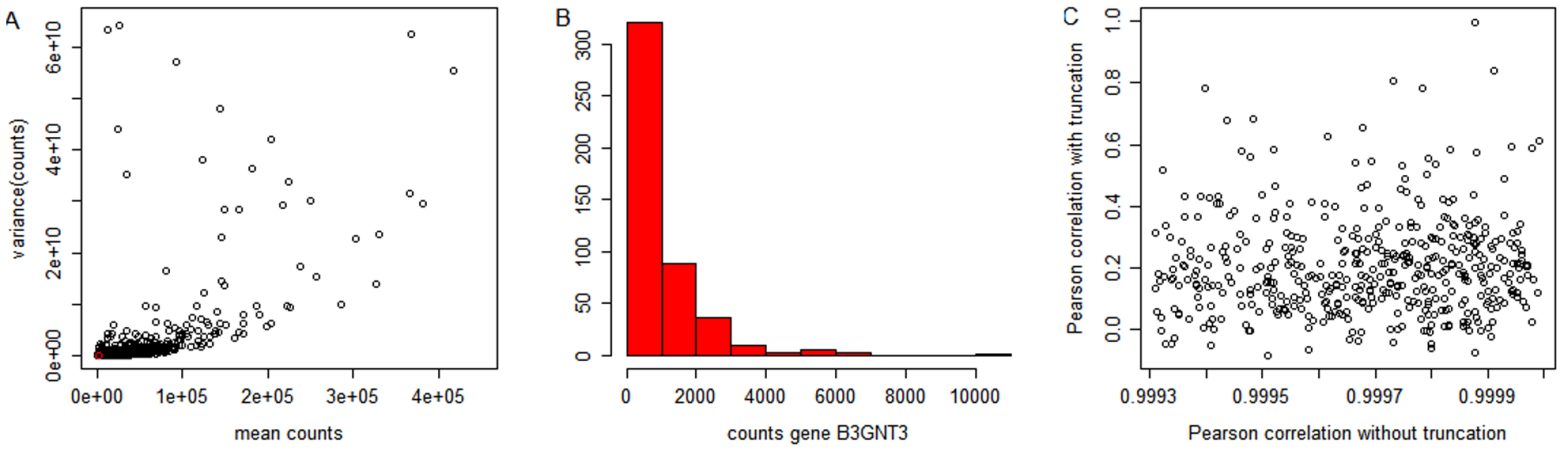
RNA-Seq of KIRC data. A: Scatterplot for all DESeq-normalized counts: Mean vs. variance. The larger the mean value, the larger the variance. The red dot is a randomly chosen gene called B3GNT3. B: Histogram of DESeq-normalized counts for gene B3GNT3. The distribution is skewed and has extreme values. C: 1000 highest gene-gene correlations for the original data compared to the same gene-gene correlations for data in which we truncated the extreme values.

Having a closer look on the RNA-Seq expression data from the KIRC patients, we can see that genes having larger mean expression values do also have larger variances (figure 1A). If we randomly select one of the genes (marked in red in figure 1A) and look at the individual expression values for this gene, we can see that they are not normally distributed but skewed (figure 1B). Both is due to the fact that RNA-Seq produces count data. Count data are known to follow a skewed distribution and have the property that the variance depends on the mean value (just remember the Poisson distribution for which the variance is exactly given by the mean). Furthermore, we can see that RNA-Seq produces some extreme values. They can be much more extreme than the one in figure 1 B. The results of multivariable modeling will critically depend on correlations between covariates. Figure 1C illustrates the effect of the extreme values on correlation. On the x-axis we have the thousand largest gene-gene correlations calculated without prior truncation and on the y-axis we have the same gene-gene correlations but calculated after truncation. We can see that extreme values lead to correlation estimates of 0.99 or larger, although there is no or even negative correlation for the truncated data. As indicated above, we use truncated measurements in this paper, to avoid problems arising from the extreme values.

### 2.2 Acute myeloid leukemia (AML) data

As a second application example, we consider RNA-Seq data from patients with acute myeloid leukemia (AML), also available from the TCGA website. There are 200 patients with clinical data available and 182 of them have RNA-Seq measurements. We excluded 13 additional patients due to unknown survival times. For the RNA-Seq expression data we followed the same preprocessing, normalization and truncation steps as for the KIRC data and end up with 169 patients and 18,714 genes. The median overall survival time for these patients is 1.3 years, 1-year and 3-year overall survival rates are estimated to be 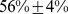 and 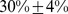. In addition, we have information on some clinical covariates including age at diagnosis and sex. For AML it is known that gene FLT3 is a very strong predictor of overall survival, see Bullinger et al. [26]. Standardization of covariates might have a different result in this setting with at least one strong signal.

## Methods

### 3.1 Effects of covariate variance in regularized regression

In this section we analytically highlight how covariates with different variances influence the model building process in regularized regression approaches. In the following we briefly describe the prominent types of regression models where regularized regression techniques are used, namely generalized linear models and the Cox proportional hazards model.

In generalized linear models, there is a response 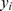, which might, e.g., be continuous, binary, or a counting response, and a covariate vector 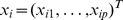, containing 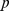 covariates, such as RNA-Seq measurements and clinical characteristics, for each patient 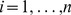. The structural part of a generalized linear model then is given by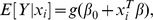 where 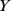 is the random variate corresponding to the response, 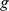 is a known response function, depending on the type of the response, 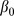 is an intercept term, and 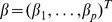 is a parameter vector of length 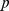, which can be estimated by maximizing the log-likelihood 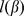 if 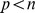.

In a time-to-event setting, observations often are given by an observed time 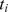, a binary variable 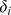 that indicates whether an event has occurred at time 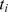, and a covariate vector 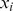. The Cox proportional hazards model is given by 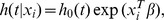
where 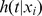 is the instantaneous risk of experiencing an event at time 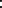 given covariate information 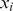 and survival up to time 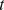. The baseline hazard 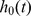 does not need to be estimated, and an estimate of the parameter vector 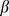 can be obtained by maximizing a partial log-likelihood, also denoted by 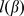 in the following, if 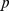 is smaller than the number of events.

In the following, we investigate the effect of differences in variance between covariates for componentwise likelihood-based boosting and penalized likelihood-approaches.

#### 3.1.1 Componentwise likelihood-based boosting

Likelihood-based boosting transfers the idea of stagewise regression [27] to generalized linear and additive models [11], [28] and to the Cox proportional hazards model [12]. At the same time, it provides a link to gradient boosting [29], which adapts a popular approach from the machine learning community.

Componentwise likelihood-based boosting starts with an estimated parameter vector 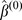 equal to zero, and updates its elements in a large number of boosting steps. In each step, candidate models are fitted, one for each covariate, and the covariate corresponding to the best candidate model is selected for an update. For generalized linear models, the candidate models in step 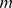 have the form of 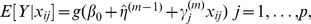
where 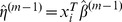 incorporates the information from the previous boosting steps, and the parameters 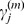 are estimated by a penalized log-likelihood 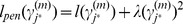. The element of the estimated parameter vector corresponding to the best candidate model 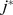 is updated by 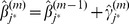. Estimates for the Cox proportional hazards model are obtained in a similar way. For both types of models, generalized linear models and the Cox model, the best candidate model can be determined by a penalized score test statistic 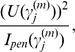
where 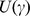 is the score function, and 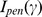 is the penalized version of the Fisher information. In a continuous response setting with orthogonal covariates, this results in estimates equivalent to those from the lasso [27]. More generally, for the continuous response setting and centered covariates, this penalized score statistic takes the form 
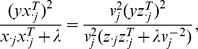
where 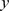 is the row vector of response, 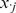 is a row vector containing all observations for covariate 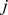, and 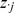 contains the standardized covariates with 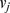 being the standard deviation of covariate 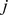. From this it is seen that scaling of covariates would cancel out if the penalty parameter 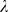 in the denominator was equal to zero. For non-zero 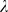, covariates with larger variance receive a smaller penalty, resulting in larger score statistics and therefore likely selection and larger updates. This has two effects: First, genes with large variances will be selected more often. Second, for the selected genes with large variances there will be less shrinkage of the parameter estimate compared to genes with smaller variances. The first aspect is much more important in high-dimensional data settings, because we are often more interested in selecting differentially expressed genes than in the estimates themselves. However, the parameter estimates gain importance in settings in which we build prognostic signatures to predict patient outcomes.

In a continuous response setting, i.e. a generalized linear model with identity link, componentwise likelihood-based boosting is equivalent to stagewise regression [27]. The latter can provide solutions similar to the lasso, which even are equivalent when infinitesimally small steps are used in a setting with orthogonal covariates. This indicates that the variance dependence of componentwise likelihood-based boosting also transfers to the lasso.

#### 3.1.2 Ridge regression

While componentwise likelihood-based boosting or the lasso provide sparse solutions, ridge regression [30] provides regularized estimates without variable selection, i.e. non-sparse solutions. In the following, we illustrate the variance dependence for such kinds of regression modeling approaches. Standardization of covariates, resulting in mean zero and variance one for every covariate, has been recommended for application of the ridge, and some of the implementations perform this as a default. However, there are some authors which explicitly do not use standardization in the context of microarray gene expression data [31]. Efficient algorithms to compute the estimates especially in high-dimensional settings have become available recently, see e.g. Goeman [32].

For simplicity let us now consider a continuous response and orthogonal, centered covariates with variance 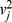, and 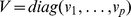 being a diagonal matrix of the standard deviations. Then the unstandardized 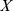 can be represented by 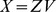 with 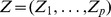 being the standardized covariates having zero mean and variance one for all 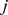. In the ordinary linear regression without regularization and 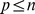 the least-squares estimates 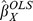 are just an 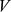-multiple of the least-squares estimates 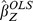 of the standardized covariates 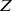: 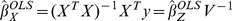



Correspondingly, the parameter estimates are independent of rescaling covariates.

For obtaining estimates in the case 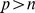, ridge regression [30], [33] attaches a penalty term of the form 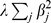 to the (partial) log-likelihood 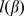. In ridge regression we minimize the penalized residual sum of squares 
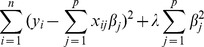
and in the case of 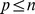 we obtain a closed form estimate for the standardized variables 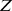: 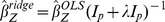



For the unstandardized covariates 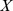 we obtain 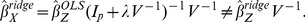
(1)


In the special case that the variances for the unstandardized covariates 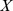 are all equal, 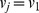 for all 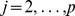, we have 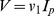. Then, in equation (1), the first occurrence of 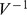 can be absorbed into the penalty term 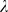. If we use the penalty 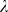 for the standardized 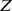
′s and the penalty 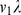 for the non-standardized 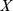
′s we will arrive at 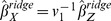, leading to the result for the ordinary least-squares estimates. Going back to the more general case of covariates 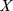 having unequal variances with 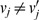 for 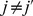, we can still absorb the diagonal matrix 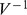 into the penalization term 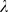, but this leads to individual penalty terms 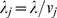 which depend on the variances of the individual covariates. Hence, covariates having larger variances will be penalized to a smaller degree than covariates with smaller variances. This results in the ridge regression preferring covariates having large variances.

### 3.2 Transformations of RNA-Seq data

**Figure 2:**
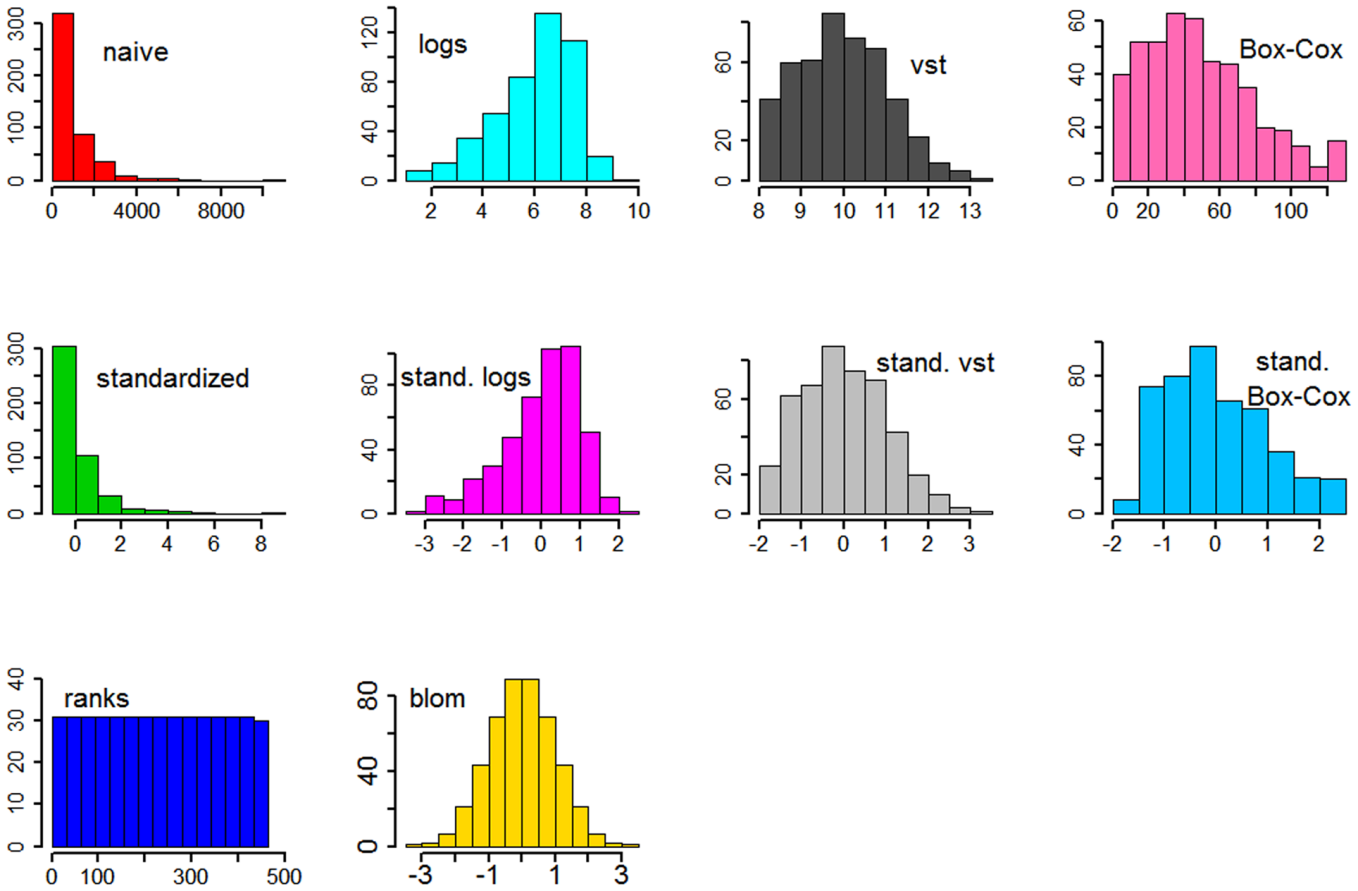
Transformed expression data for gene B3GNT3.

As seen in the KIRC data, data coming from RNA-Seq have three problematic properties, namely a skewed distribution, unequal variances for the individual genes and the presence of extreme values. In the following we propose to transform RNA-Seq data before applying a regularized regression approach to potentially deal with all these properties. We compare the use of the untransformed data with several different transformations. The transformations can be separated into three classes: transformations not including standardization of covariates, transformations including standardization of covariates and non-parametric transformations. Each of the transformations tries to handle either one, two or all of the three problematic properties. Table 1 gives a summary of all transformations considered and Figure 2 gives a histogram for the gene expression of gene B3GNT3 for each of the considered transformations.

**Table 1:** Transformations for RNA-Seq data.

Transformation	Skewness	Unequal variances	Extreme values
Naive	–	–	–
Logs	(**√**)	–	**√**
Variance stabilizing	–	(**√**)	**√**
Box-Cox	(**√**)	–	**√**
Standardizing	–	**√**	–
Standardizing logs	(**√**)	**√**	**√**
Standardizing variance stabilizing	–	**√**	**√**
Standardizing Box-Cox	(**√**)	**√**	**√**
Ranks	**√**	**√**	**√**
Blom	**√**	**√**	**√**

Proposed transformations for RNA-Seq data. A check mark is given in the columns skewness, unequal variances or outliers, if the transformation is addressing the corresponding problem. The last column shows the transformed distribution of gene B3GNT3 as an example.

#### 3.2.1 Naive analysis

The naive way of analyzing RNA-Seq data in regularized approaches is to use the normalized counts without further transformation. We will call the normalized, but untransformed counts 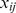. For the 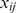 we have skewed distributions, unequal variances and some extreme values.

#### 3.2.2 Log transformation

In ordinary regression analysis, the log transformation is often used for covariates with skewed distribution, and so might also be useful for RNA-Seq data. The log-transformed data are expected to be more or less normally distributed, depending on the degree of skewness before transformation. As the normalized counts 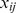 can be equal to zero, we shift them by one before log-transforming them, i.e. 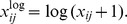



For RNA-Seq data this does not lead to perfectly shaped normal distributions (see figure 2), but the distribution is typically less skewed than before transformation. The log-transformed data have less extreme values compared to the untransformed data, but they still have unequal variances for the covariates.

#### 3.2.3 Variance-stabilizing transformation

Anders and Huber [14] proposed a variance stabilizing transformation for RNA-Seq data, which is implemented in the R package DESeq. Variance stabilizing transformations are used to obtain covariates with variances independent of the mean value. Anders and Huber model the relationship between the mean expression values 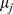 and the variances 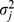 by 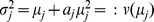, with 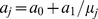 being a dispersion parameter and 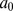 and 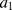 are estimated in a generalized linear model. Details can be found in the vignette of the R-package. The variance-stabilized expression values can be calculated using the modeled mean-variance relationship 
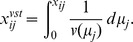



The variances for the transformed data are approximately independent of the mean value, but they are still unequal for all genes. The variance stabilized counts have a less skewed distribution but may include extreme values.

#### 3.2.4 Box-Cox transformation

The Box-Cox transformation is a class of power transformations which has been developed to transform data in such a way that they satisfy the normality assumption [34]. The Box-Cox transformed gene expression values are defined as 
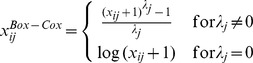
where 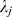 is a tuning parameter for gene 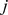 that can be optimized in a way that the distribution of the transformed data has the largest similarity to a normal distribution. There are several proposals to optimize 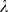, see e.g. [35], we have chosen the optimality criterion as a maximum Pearson correlation within the QQ-plot for the transformed data. We optimize 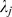 for gene 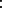 as a multiple of 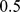 within the interval 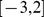. For all 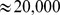 genes of the KIRC and the AML data, 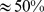 of the times 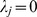 and therewith the log transformation has been chosen, 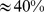 of the 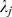
's were equal to 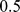 or 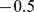 and 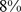 of the times the algorithm has chosen a 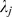 smaller than 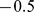. For only 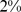 of the genes, the algorithm led to a value of 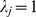 for which the Box-Cox transformation equals the identity. These 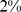 were mainly very high expressed genes having no extreme values. The Box-Cox-transformed expression values do not guarantee normality although the data should be less skewed and should have less extreme values than before transformation.

#### 3.2.5 Standardization

Standardizing the covariates is the default implementation in many regularized regression techniques. A gene-wise standardization of the expression values contained in a covariate 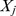 is obtained by: 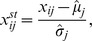
with estimated mean value 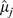 and estimated standard deviation 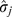. This transformation leads to empirical zero mean and variance one for each gene 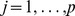. The distributions of the standardized covariates retain their skewness and still might include extreme values. The degree of skewness is different for different genes.

#### 3.2.6 Standardized logs

The standardized log transformation is a combination of log transformation and standardization. We standardize the log-transformed values by their estimated mean 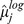 and standard deviation 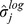, 
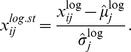



Again this does not result in perfect normal distributions, but the transformed data typically are less skewed, have less extreme values and have exactly mean zero and variance one for all genes. The standardized logs therefore potentially address all three problematic properties of RNA-Seq data.

#### 3.2.7 Standardized variance-stabilizing transformation

The standardized variance-stabilizing transformation is a combination of the variance-stabilizing transformation and standardization. We standardize the variance-stabilized values by their estimated mean 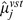 and standard deviation 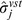: 
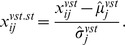



This leads to empirical mean zero and variance equal to one for all genes.

#### 3.2.8 Standardized Box-Cox transformation

The standardized Box-Cox transformation is a combination of Box-Cox transformation and standardization. We standardize the Box-Cox transformed values by their estimated mean 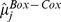 and standard deviation 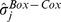, 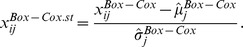



The standardized Box-Cox transformed data are less skewed, have less extreme values and have exactly mean zero and variance one for all genes.

#### 3.2.9 Ranks

Working with ranks is a simple and popular method used in non-parametric statistics. Correspondingly, we consider 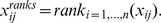



The ranks are uniformly distributed from zero to the sample size 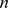. Hence, the ranks lead to exactly the same distribution for all genes, which directly leads to exactly equal means and variances for all genes. There are no extreme values in the transformed data. This transformation potentially addresses all considered problems of RNA-Seq data, although the resulting distribution is not normal. For genes with very low expression, i.e. with many zero counts, a small noise term 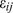 might be added before data transformation to handle the ties.

#### 3.2.10 Blom transformation

Recently, the Blom transformation has been used in genetic association studies [36]. The Blom transformation is a rank-based transformation, which back-transforms the uniformly distributed ranks to a standard normal distribution, i.e. 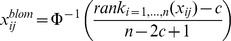
with c = 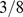. The Blom transformed data have a standard normal distribution, which results in empirical mean zero and variance one for all genes. There are no extreme values in the transformed data. The difference to the rank transformation is just the type of resulting distribution. For a gene with very low expression, i.e. with many zero counts, a small noise term 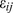 might be added to handle the ties.

### 3.3 A simulation study

In order to compare the behavior of the transformations under realistic assumptions, we decided to perform simulations with covariate structure based on a real RNA-Seq dataset, specifically the KIRC data described above. So we do not need to specify an underlying distribution for the RNA-Seq data, which may in reality fit neither poisson nor negative binomial. Based on the real RNA-Seq data we simulate a binary patient outcome, which will be described in more detail in the following.

We included the real RNA-Seq expression measurements 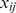 of all 465 patients of the KIRC data set and all 19,227 genes. Apart from the preprocessing and normalization steps described above, we did not change any of the RNA-Seq measurements. So we have skewed RNA-Seq data with a mean-variance-dependency not relying on any specific distribution. Furthermore, the genes are correlated and include a realistic number and size of extreme values. The real clinical data and survival times have not been used in the simulation study.

To simulate a binary outcome 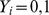 for each patient 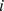, we calculated the linear predictor for each patient 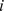 as 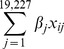
(2)where 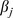 denotes the true effect of gene 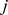 on the simulated outcome. As we assume sparsity, we randomly selected only a subset 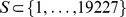 of size 10 (in a second scenario 20) different genes to have non-zero effects 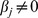. The non-selected genes have no impact on the outcome and therewith 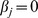 for 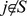. In order to have the informative genes over the whole range of gene lengths, we first ordered the genes according to their length and then divided them in either 10 or 20 equally large bins. In every bin we randomly selected one gene to be the one with impact on the outcome and so to have a non-zero true parameter value 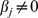. We have chosen equal effect sizes in the scenario with 10 informative genes, 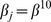 for all 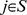, and have chosen 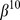 to result in an approximate signal-to-noise ratio of 2.5. The signal-to-noise ratio was defined as 
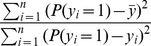
with 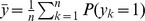. In the second scenario, in which we assumed 20 informative genes, we again assume equal effect sizes 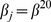 for all 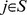 and a signal-to-noise-ratio of 2.5. This results in more genes having smaller individual effect sizes 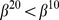, although the overall signal-to-noise ratio is maintained.

In a first run of simulations we assumed that the DESeq-normalized counts 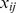 have a linear effect on the patient outcome, as indicated in the linear predictor in equation (2). Using the logistic regression model we calculated the probability to be a case for patient 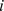: 
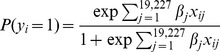



To end up with a binary patient outcome we used a bernoulli-distributed random variable with probability 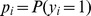 to decide if patient 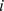 will be a case (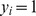) or will be a control (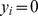). After this procedure we have real RNA-Seq data and a simulated binary patient outcome depending on a known subset of genes 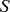. We repeated the choice of the subset of informative genes 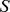 50 times and obtained 50 datasets of the matrix with RNA-Seq data and the vector of case/control-indicators 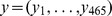 for analysis.

For each of the 50 data sets, a logistic regression model is fitted by componentwise likelihood-based boosting. As covariates for this regression model, we consider the DESeq-normalized counts in the model without transforming them further (naive analysis), and all other transformations indicated above. For the ranks and the Blom-transformation we added a small 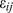 before transformation to handle the ties. 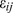 was drawn form a normal distribution with mean zero and standard deviation 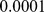. We used the penalty term 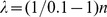 which roughly corresponds to the factor 0.1 typically used in gradient boosting. All models were built up to 500 boosting steps.

In a second run of simulations, we assumed a logarithmic effect of the gene expressions 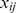 on the patient outcome. In this case, the linear predictor in equation (2) was calculated using the log-transformed counts 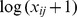: 
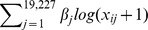



The influence of outliers is decreased in this logarithmic setting, which may seem to be reasonable.

## Results

### 4.1 Simulation study

**Figure 3:**
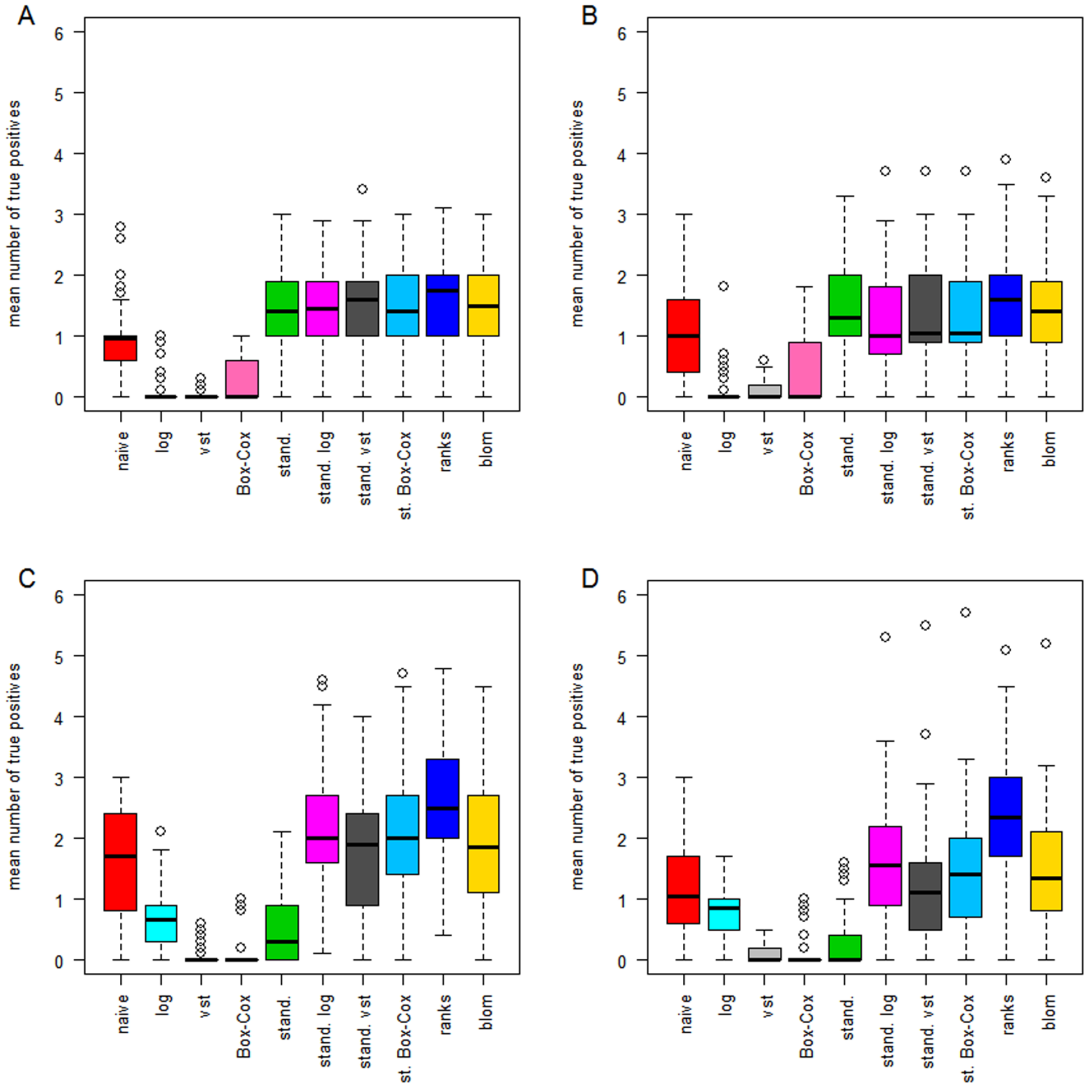
Areas under the curve for the simulation study. A: 10 genes have a linear effect on the patient outcome. B: 20 genes have a linear effect. C: 10 genes have a logarithmic effect. D: 20 genes have a logarithmic effect.

For all 50 simulation runs we calculated a ROC-like curve in which we have the number of false positive genes on the x-axis, i.e. genes with true parameter equal to zero that have nevertheless been assigned a non-zero estimate by the boosting approach, and the number of true positive genes on the y-axis. The area under this curve from zero up to ten false positive genes can be interpreted as the mean number of true positives within ten or less false positive genes. Figure 3 displays the area under the curve for all transformations. The panel at the top (A and B) provides results from the scenarios where the DESeq-normalized counts have a linear effect on the outcome while the panel at the bottom (C and D) provides the results from scenarios with logarithmic true effect in the linear predictor of the generating logistic regression model. The left panel of Figure 3 indicates the results from scenarios with 10 informative genes, the right panel with 20 informative genes.

In the scenarios with a linear effect, the four transformations that do not include standardization (naive, logarithmic, variance-stabilizing and Box-Cox) have overall worse performance than the transformations including standardization and the non-parametric ones. The variance stabilizing transformation which tries to capture and make use of the distribution of the underlying data, performs worse than the naive analysis, which may be explained by the fact that it tries to estimate a gene's variance using information of similar genes. This is helpful in settings with very low sample sizes but in the setting of prognostic gene signatures, in which one normally has plenty of samples, this will lead to more biased results than estimating the variance just using the information of the particular gene. Standardizing means and variances increases the mean true positive rate, regardless of the specific transformation by which the standardization of variances has been achieved. Standardization via the variance stabilizing transformation is not performing better than standardization of the original scale or the log-scale and the non-parametric transformations achieve similar performance compared to the approaches using standardization. Increasing the number of informative genes from 10 to 20, while not changing the signal-to-noise ratio, does not affect the overall performance or the ranking of the transformations.

The bottom panels of Figure 3 indicate the area under the curve for the scenarios with logarithmic effects of the RNA-Seq data on the patient outcome. The overall performance is better compared to the scenarios with linear true effects, although the signal-to-noise ratio is the same. Logarithmic true effects decrease the influence of extreme values and this seems to result in a less difficult modeling problem. Interestingly, standardizing the original scale of the covariates, which is the default of most implemented regularized regression techniques, performs poor. This is because this transformation mis-specifies the association between expression data and outcome. This results in performance that is even worse than the naive analysis. The rank-based transformations perform best, followed by standardization of the logs. Standardization of the Box-Cox transformed data, which led to 50% of the genes being log-transformed and 50% other power-transformations, can not outperform standardization of the log-transformation of all genes.

Generally, the results of the simulation study show that standardizing the expression data has a large effect on performance with respect to genes that truly have an effect, although different underlying assumptions of the effects result in other orderings of the performances of the transformations. The performance of the standardized variance-stabilizing approach, which is not better than using standardization of the log-scale, may furthermore imply that RNA-Seq data are much more complex than Poisson- or negative binomial distributions or that the complex re-parametrization is not fully capable of handling the extreme values. Furthermore, we find that the robustness of the rank-based transformations cannot only compensate the lack of all distributional assumptions, but can even outperform all other transformations in some scenarios.

### 4.2 Application to real data: KIRC and AML

In the following we compare the transformations on the real datasets of patients with kidney renal clear cell carcinoma and acute myeloid leukemia introduced above. One main question is whether the RNA-Seq data include additional information concerning the survival times of the patients beyond the clinical covariates. We analyzed the clinical covariates using forward and backward Cox regression. For the KIRC data, four clinical covariates showed an impact on the overall survival time, which were age at diagnosis (continuous), laterality (the right or left kidney), tumor stage (I vs. II vs. III vs. IV) and platelet count (elevated vs. normal vs. low). For the AML data, two of them showed an impact on the overall survival time, which were age at diagnosis (continuous) and sex (female vs. male). These clinical covariates were included as mandatory and unpenalized covariates in a Cox model fitted either by the lasso [37] or by componentwise likelihood-based boosting [12]. The RNA-Seq data of the 19,227 genes for the KIRC data and 18,714 genes for the AML data were added optional and thus as regularized covariates. To obtain risk prediction models for evaluating prediction performance, 10-fold cross-validation was used to determine the optimal penalty parameter 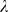 for the lasso and the optimal number of boosting steps for componentwise likelihood-based boosting.

For more detailed evaluation, we use a resampling procedure, i.e. we randomly split the data in training and test set for a number of 50 times, drawing 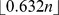 observations without replacement for each respective training set, and retaining the others for test sets. Model fitting, including selection of the number of boosting steps, was performed in each of these training data sets. Evaluating selection for each gene across the resampling data sets then allows to better judge the effect of different transformations, compared to evaluation on a single data set. Furthermore, resampling data sets allow to estimate the prediction performance for new observations. Specifically, we consider 0.632+ prediction error curves for judging prediction performance over the course of time [38].

To quantify the added value of a model combining RNA-Seq and clinical data compared to a clinical model, we consider integrated prediction error curves (IPEC), i.e. the area under the prediction error curve. Thus, added value in the 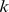
th test data is given by 
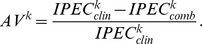



This measure of added value is zero if there is no improvement in the prediction error, and it is negative if the prediction performance of the combined model is worse than that of the clinical model. For combined models which improve the prediction performance the added value is in 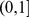 and can be interpreted as the proportion of prediction error of the clinical model that could be eliminated using the combined model.

To have a closer look on the characterization of selected genes, we build up a model up to 200 steps for the KIRC data. The models with 200 boosting steps will certainly be overfitted, i.e. will include too many genes, but this will allow more stable characterization of the selected genes, e.g. when considering their median variance. We used the DESeq-normalization before transforming the data.

#### 4.2.1 Characterization of selected genes in the KIRC data

Table 2 shows means and standard deviations of the number of selected and overlapping genes in 50 resampling datasets in which we used componentwise likelihood-based boosting with 200 steps. We can see that the overlap between the four transformations not including standardization (naive, log, variance-stabilizing, Box-Cox) is very small: less than 1 out of 40-100 genes has been selected by all four transformations. The four transformations using standardization (of the original scale, of the log-scale, of the variance-stabilized data or of the Box-Cox-transformed data) select approximately 65 genes and have a larger overlap of approximately 4 selected genes. The two rank-based methods select approximately 80 genes and have an overlapping proportion of about one third. This may imply that standardization itself is more important than the exact distribution used for standardization. The overlap between the rank-based transformations and those using standardization is larger than the overlap between the rank-based ones and those not using standardization (1.9 vs 0.0).

**Table 2:** KIRC data: Number of selected genes in a CoxBoost model of the KIRC data.

	non-stand.					stand.					non-par.		
	naive	log	vst	Box-Cox	all	st.	st.log	st. vst	st. Box-Cox	all	ranks	blom	all
naive	113.9												
	(4.9)												
log	1.3	44.5											
	(1.3)	(3.9)											
vst	7.0	9.3	38.3										
	(1.9)	(2.4)	(3.1)										
Box-Cox	5.8	0.1	1.1	116.0									
	(2.0)	(0.4)	(0.8)	5.5)									
all					0.1								
					(0.3)								
stand.	2.0	7.3	3.2	0.9		65.6							
	(1.3)	(2.5)	(1.8)	(0.8)		(5.4)							
stand. log	1.9	9.9	3.7	0.3		26.1	65.0						
	(1.1)	(2.7)	(1.8)	(0.5)		(3.4)	5.9)						
stand. vst	2.0	8.4	4.0	0.3		27.0	40.0	66.6					
	(1.5)	(2.6)	(4.3)	(0.5)		(3.5)	(4.3)	(6.2)					
st. Box-Cox	0.9	3.0	1.2	1.3		7.2	8.1	7.6	64.6				
	(1.0)	(1.6)	1.1)	(1.0)		(2.8)	(2.8)	(2.9)	(5.1)				
all					0.0					4.1			
					(0.0)					(1.9)			
ranks	2.1	7.4	2.9	1.2		21.8	27.1	22.8	8.5		74.8		
	(1.2)	(2.3)	(1.5)	(0.9)		(3.4)	(4.1)	(4.0)	(3.4)		(4.8)		
blom	1.3	5.6	2.0	0.8		13.8	16.8	15.3	6.8		24.9	94.2	
	(1.1)	(2.0)	(1.4)	(0.9)		(3.6)	(3.19)	(3.2)	(2.4)		(4.4)	(5.6)	
all					0.0					1.9			24.9
					(0.0)					(1.5)			(4.4)

The transformations are separated in three blocks: those not using standardization, those using standardization and the non-parametric ones. The diagonal elements give mean and standard deviation for the corresponding transformation. Diagonal elements called “all” give the number of overlapping genes for the whole block of transformations. Non-diagonal elements show the number of overlapping genes for two transformation, the non-diagonal elements called “all” give the overlap between two blocks.

**Figure 4:**
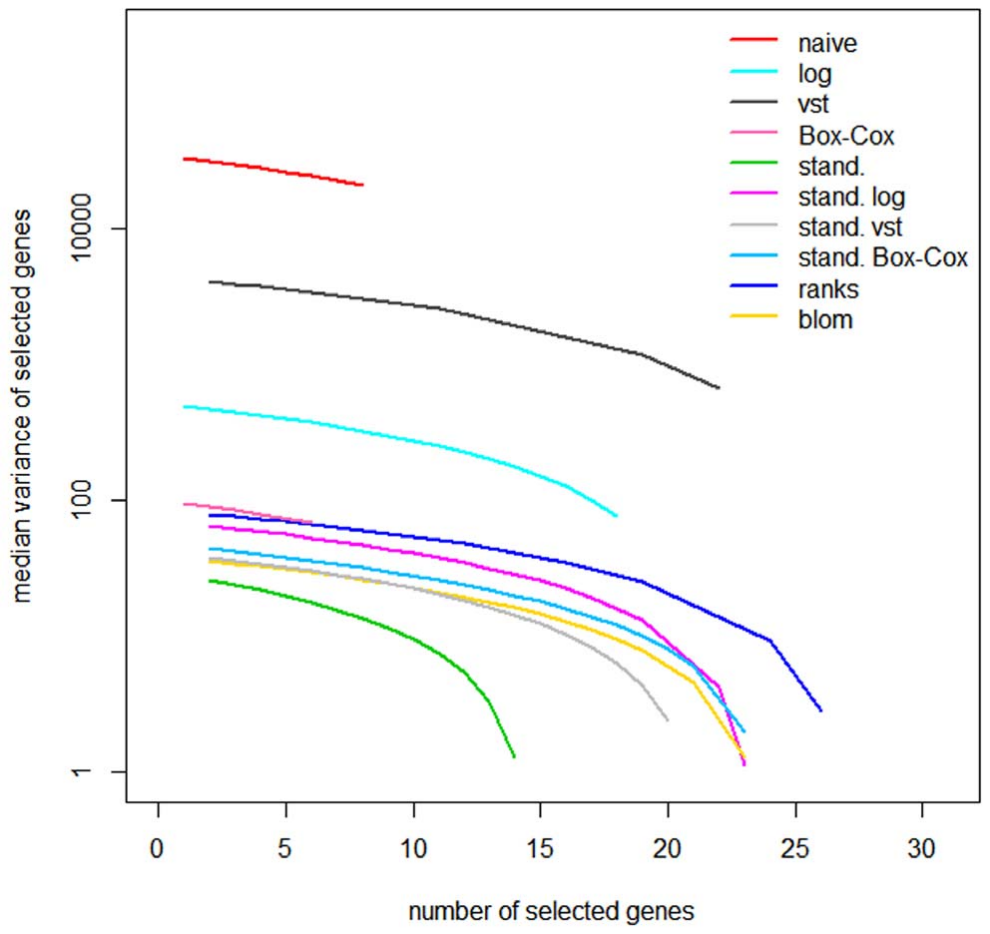
Selected genes in the KIRC data. Median variance of selected genes plotted against the number of selected genes in 50 resampling datasets. We used a smoothing spline on the scatterplot for better visualization of the association.

Figure 4 shows the number of selected genes plotted against the median variance of the selected genes in 50 resampling datasets, where the number of boosting steps was selected via 10-fold cross-validation. We can see that in a model which already includes some genes with large variances, the boosting algorithm stops early and the final model includes a small number of genes. In a model where the majority of genes have small variances, the algorithm proceeds and allows a larger number of genes to be included in the final model. Such a pattern is seen for all the transformations, although the transformations not using standardization generally incorporate genes with larger variances over the whole range of numbers of selected genes.

#### 4.2.2 Prediction performance in the KIRC and AML data

**Figure 5:**
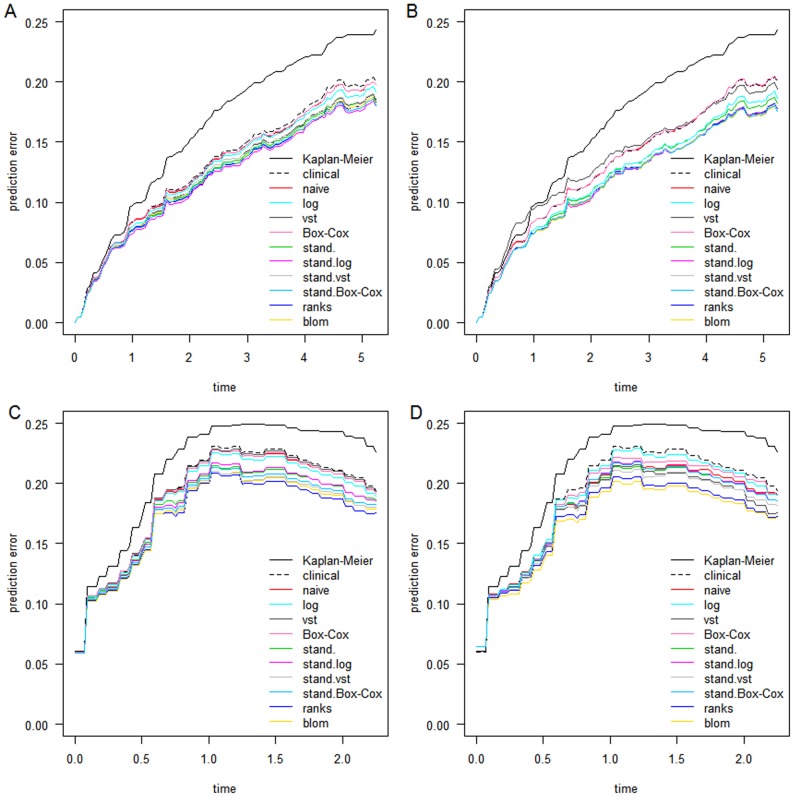
Prediction error for the KIRC and AML data. The 0.632+ estimator for the prediction error in terms of the Brier Score. The solid black line is the prediction error of the Kaplan-Meier estimate which does not include clinical information nor RNA-Seq data, the dashed black line the prediction error of the clinical model. A: CoxBoost model used for prediction on KIRC data. B: Lasso used for prediction on KIRC data. C: CoxBoost model used for prediction on AML data. D: Lasso model used for prediction on AML data.

The 0.632+ prediction error curve estimates, calculated over all 50 resampling data sets, is shown for each of the proposed transformations in Figure 5. The upper panel gives the prediction error for the KIRC data and the lower panel for the AML data. The results of the componentwise likelihood-based boosting approach are shown in the left panel and the results for the lasso in the right panel. In each of the four figures the black solid line is the prediction error for the Kaplan-Meier estimate which takes neither the clinical covariates nor the RNA-Seq data into account, while the black dashed line is the prediction error curve for the model including only the clinical covariates. In nearly all cases, the prediction error curves for all transformations are seen to be below the prediction error curve for the clinical model, indicating added value over the clinical model. The degree of improvement depends on the chosen transformation. Similar to the results of our simulation study, the transformations not using standardization all have larger prediction errors than their standardized counterparts, for both datasets and both model building approaches. Thus, choosing a small number of genes having large variances does not seem to have an advantageous effect on prediction performance in both real datasets. For the AML data we assumed at least one gene to have a large effect on overall survival, so that these results may not depend on the true signal sizes of the underlying data. Standardization without using any further data transformation shows the overall worst performance within the transformations using standardization. The ordering of the remaining transformations using standardization is small and seems to depend on the dataset: Standardization of the logs has the best performance in the KIRC data and the worst in the AML data. The rank-based transformations perform well for the KIRC data and very good for the AML data, for both the boosting approach and for the lasso.

**Figure 6:**
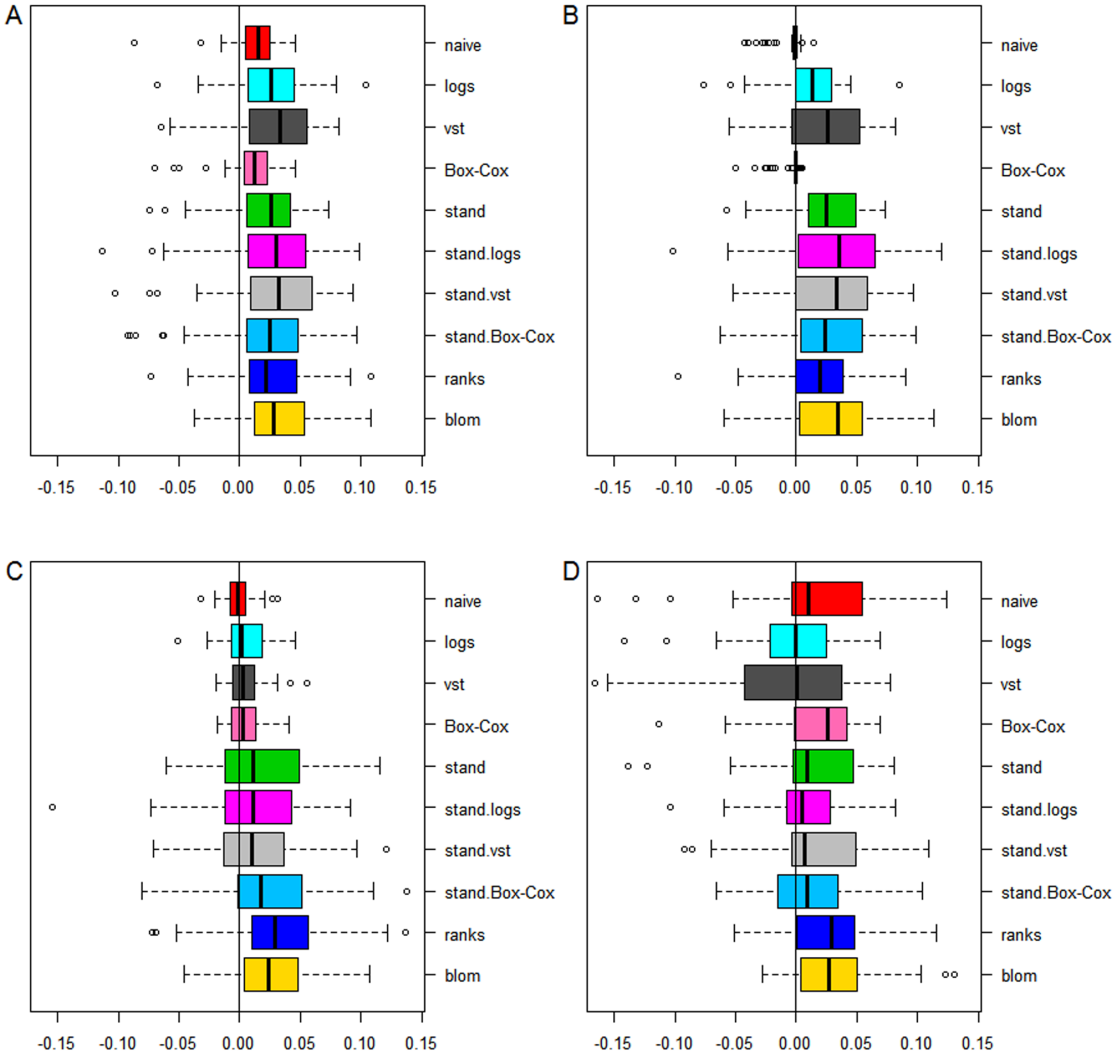
Added value for KIRC and AML data. Positive added values indicate improvement in prediction error. A: CoxBoost model used for prediction on KIRC data. B: Lasso used for prediction on KIRC data. C: CoxBoost model used for prediction on AML data. D: Lasso model used for prediction on AML data.

Figure 6 shows the added value 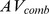 of the models combining RNA-Seq data with clinical data compared to a solely clinical model for the 50 splits in training and test data. The upper and lower panel again give the results for the KIRC data and the AML data, while the left and right panel display the results for boosting and the lasso. The transformations using standardizations as well as the rank-based ones have added value compared to the model only including the clinical variables in all four application examples. The added value of the transformations not using standardizations is either very low or cannot be seen at all. We can see that the variances of the added value differ for the transformations: The transformations not using standardization seem to have smaller variances in the boosting approach, while this cannot be seen clearly in the lasso.

## Discussion

For high-dimensional data arising from RNA-Seq many univariable testing procedures have been developed and implemented within the last five years [13]–[20]. In contrast, there is only little guidance available for multivariable modeling with RNA-Seq data. This is a pity, as in particular regularized regression techniques can be applied to select a small and manageable number of differentially expressed genes, while at the same time directly providing predictions for new patients.

As regularized regression techniques depend on the covariates variances and may more generally be critically affected by covariate distributions, we have compared different transformations of RNA–Seq data in terms of signature size, identification of important genes, and prediction performance. For gene expression measured by microarrays, it has been argued that no standardization is needed, as the measurements already are on the same scale [31]. However, we focus on criteria such as identification and prediction performance that nevertheless might make standardization attractive for RNA-Seq data, despite having measurements at the same scale.

First, we used a two-group simulation study with covariate structure based on real RNA-Seq data. We included scenarios with a few genes having large signals and scenarios with a larger number of genes having smaller signals. Transformations that result in equal variances for all individual genes were seen to perform better than those not standardizing variances. The performance of the rank-based transformations is consistently competitive in many different scenarios, which, e.g., was not the case for the default of standardization. Also, complex variance-stabilizing approaches did not outperform the rank-based approaches. This is in line with the comparison of univariable testing procedures for RNA-Seq data of Soneson et al. [22], who found that the nonparametric SAMSeq method [16] works quite well in situations with at least ten samples per group.

Second, we could show in an application to two different real datasets consisting of patients with kidney renal clear cell carcinoma and acute myeloid leukemia that the transformations behave similar in real data with time-to-event outcomes. Standardization of covariates leads to better prediction performance independent of the underlying transformation used (original scale, log-scale, VST, Box-Cox) and independent of the underlying regression model (componentwise likelihood-based boosting and the lasso). The exact type of transformation used has a smaller effect on prediction performance than standardization and its effect seems to depend on the real dataset. The number of selected genes and their variances depend highly on the transformation used. There is a large overlap of selected genes for the transformations including standardization and a small overlap for all other transformations.

The results of this study suggest that transforming the data to a distribution with equal variances for all genes is an important step if RNA-Seq data are going to be analyzed in regularized regression. The choice of a suitable transformation is essential and has a large influence on the genes being selected as differentially expressed, on the number of true positives and on the prediction performance of the model. While these results are supported by a simulation study with different numbers of important genes, i.e. sparse and non sparse scenarios, and two real data sets, this naturally does not guarantee generalizability to other data sets. However, the results at least point out that transformation and standardization are important issues that need to be carefully considered as a part of modeling, as they can have a strong detrimental effect on performance for different kinds of multivariable regression approaches. We expect that these results are not specific for 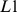-penalized regression models like componentwise likelihood-based boosting and the lasso, but will equally apply to the ridge regression, as indicated in an analytical part on the effect of covariate variance. Also, other types of regularized regression, e.g. the elastic net, might be affected in a similar way.

While covariate variance and more generally covariate distribution, also might affect performance for other molecular platforms, the pattern might not necessarily be the same as for RNA-Seq data. For single nucleotide polymorphism data an investigation found even somewhat better performance for covariates without standardization [24]. Other molecular platforms, e.g. DNA methylation, might show still other patterns. Further research will be necessary to decide the best fitting transformation for each of the different platforms.
